# Comparing Gastrectomy Complications Consensus Group (GCCG) and Esophagectomy Complications Consensus Group (ECCG) Classifications in Reporting Postoperative Complications After Gastrectomy: A Population-Based Nationwide Study in Finland

**DOI:** 10.1245/s10434-025-17674-2

**Published:** 2025-06-23

**Authors:** Emilia Putila, Olli Helminen, Mika Helmiö, Heikki Huhta, Aapo Jalkanen, Anna Junttila, Raija Kallio, Vesa Koivukangas, Arto Kokkola, Elina Lietzen, Johanna Louhimo, Sanna Meriläinen, Vesa-Matti Pohjanen, Tuomo Rantanen, Ari Ristimäki, Jari V. Räsänen, Juha Saarnio, Eero Sihvo, Vesa Toikkanen, Tuula Tyrväinen, Antti Valtola, Joonas H. Kauppila, the FINEGO Group

**Affiliations:** 1 Surgery Research Unit, Medical Research Center Oulu, Oulu. University Hospital and University of Oulu, Oulu, Finland; 2 Division of Digestive Surgery and Urology, Turku University Hospital, Turku, Finland; 3 Department of Surgery, University of Helsinki and Helsinki University Hospital, Helsinki, Finland; 4 Department of Oncology and Haematology, Oulu University Hospital, Oulu, Finland; 5 Cancer and Translational Medicine Research Unit, Medical Research Center Oulu, University of Oulu and Oulu University Hospital, Oulu, Finland; 6 Department of Surgery, University of Eastern Finland and Kuopio University Hospital, Kuopio, Finland; 7 Department of Pathology, HUSLAB, HUS Diagnostic Center, Helsinki University Hospital and University of Helsinki, Helsinki, Finland; 8 Applied Tumor Genomics Research Program, Research Programs Unit, Faculty of Medicine, University of Helsinki, Helsinki, Finland; 9 Department of General Thoracic and Oesophageal Surgery, Heart and Lung Centre, University of Helsinki and Helsinki University Hospital, Helsinki, Finland; 10 Department of Surgery, Central Finland Central Hospital, Jyväskylä, Finland; 11 Department of Cardiothoracic Surgery, Heart Center, Tampere University Hospital and University of Tampere, Tampere, Finland; 12 Department of Gastroenterology and Alimentary Tract Surgery, Tampere University Hospital, Tampere, Finland; 13 Department of Molecular Medicine and Surgery, Karolinska Institutet and Karolinska University, Stockholm, Sweden

## Abstract

**Background:**

Previously, no international consensus on reporting complications after gastric cancer surgery existed, making comparisons between studies difficult. In 2015 the Esophagectomy Complications Consensus Group (ECCG) published a standardized list for classification of postoperative complications after esophagectomy for esophageal cancer, which also was applied for gastric cancer. In 2019 the Gastrectomy Complications Consensus Group (GCCG) reported outcomes after gastrectomy for gastric cancer with a list of different complication types. This study aimed to compare the two classifications in reporting postoperative outcomes after gastrectomy for gastric cancer.

**Methods:**

This population-based study, based on the Finnish National Esophago-Gastric Cancer Cohort, included all patients age 18 years or older undergoing gastrectomy for gastric cancer in Finland during 2010–2016. For classifying and describing different postoperative outcomes, both the ECCG and GCCG lists of complications were used separately.

**Results:**

The study analyzed 1115 patients. The occurrence of complications 90 days postoperatively was 23.0% according to the GCCG classification (504 individual complications) and 43.0% according to the ECCG classification (1084 individual complications). Most of the notable differences between the classifications in reporting complications were in cardiac dysrhythmia, infections, and myocardial infarction, with the GCCG classification reporting a lower incidence. Additionally, 131 complications occurring in 13 individual types of complications defined only in the ECCG and not in the GCCG were recorded.

**Conclusions:**

This study suggests that the ECCG classification is more comprehensive and sensitive in evaluating complications of gastrectomy than the GCCG classification. Use of the ECCG classification may be preferable in the context of gastrectomy.

Gastric cancer is the third leading cause of cancer death worldwide, with nearly 800,000 annuals deaths in 2020.^1,2^ Because early gastric cancer is often asymptomatic, most patients have an advanced stage of disease at the time of diagnosis, with a poor prognosis. Even for patients undergoing radical resection (R0), the 5-year survival rate is lower than 30%.^3^

The target surgery for gastric adenocarcinoma is a curative gastrectomy involving removal of all gross cancer and regional lymph nodes without leaving any macroscopically visible cancer lesions.^4^ Nevertheless, gastrectomy for gastric cancer still has high complication and mortality rates.^5^

Previously, no international consensus on reporting complications after gastrectomy for gastric cancer existed, making international comparisons difficult. Several publications use the Clavien-Dindo classification^6^ for reporting the severity of the complications, but it does not differentiate the various types of postoperative complications.

In 2015 the Esophagectomy Complications Consensus Group (ECCG) published a standardized list for classification of postoperative complications after esophagectomy for esophageal cancer, aiming to standardize the international outcome reporting.^7^ Because an international standardized outcomes set for gastrectomy was lacking during the inception of the Finnish National Esophago-Gastric Cancer Cohort (FINEGO) study, the ECCG outcomes set was applied for patients undergoing gastrectomy for gastric cancer due many similarities in the surgical approaches and their complications.

Gastrectomy and esophagectomy for gastric and esophageal cancer have comparable types and severities of postoperative complications, and the Dutch Upper Gastrointestinal Cancer Audit (DUCA) group successfully reported outcomes after gastrectomy using the ECCG classification in a nationwide cohort setting.^5^ The ECCG upper-level categories are pulmonary, cardiac, gastrointestinal, thromboembolic, neurologic, urologic, infectious, wound, and other complications. The lower-level categories consist of 48 specific complications under the upper-level categories.

In 2017 the Italian Research Group for Gastric Cancer (CIRCG) published a list of 13 different complications with definitions after gastrectomy for gastric cancer.^8^ Later, in 2019, the Gastrectomy Complications Consensus Group (GCCG) published a list of 27 different complication types including 3 intraoperative, 14 general postoperative, and 10 surgical postoperative complications, aiming to provide a list of gastrectomy-specific complications with definitions and with international consensus.^9^ According to the GASTRODATA registry, the GCCG classification was used for evaluating outcomes after gastrectomy for gastric cancer.^10^

The current study aimed to compare these two classifications (GCCG and ECCG) on reporting postoperative complications in a population-based setting. The objective was to observe whether the two classifications result in different rates of postoperative outcomes using the same cohort data, and to point out which types of complications are underreported by each instrument in the context of gastric cancer.

## Methods

### Study Design

This population-based nationwide cohort study was based on the Finnish National Esophago-Gastric Cancer Cohort (FINEGO), which includes patients with gastric cancer or a tumor diagnosis in the Finnish Cancer Registry or the Finnish Patient Registry. This study was based on patients who underwent gastrectomy in Finland during 2010–2016. The inclusion criteria specified a diagnosis of gastric adenocarcinoma and an age of 18 or older at the time of diagnosis. The data collection is described in previous publications.^11,12^ For the purpose of this study, the patient records were re-reviewed to evaluate the complications according to the definitions of the GCCG.^9^

### Definitions of Complications

The GCCG classification of complications after gastrectomy for gastric cancer consists of 27 different complication types, of which 3 are intraoperative and 24 are postoperative.^9^ The 24 different postoperative complications are divided into 14 general and 10 surgical postoperative complications. In the current study only postoperative complications were assessed.

The ECCG classification of complications consists of nine different upper-level categories: pulmonary, cardiac, gastrointestinal, thromboembolic, neurologic, urologic, infectious, wound, and other complications.^5^ The ECCG upper-level categories consist of lower-level categories, which are specific complications grouped under the upper-level categories. The ECCG classification comprises 48 specific complications in the lower-level categories.

### Outcomes

The primary aim was to evaluate whether the classifications GCCG^10^ and ECCG^5^ differ from each other in reporting postoperative complications after gastrectomy for gastric cancer. The secondary aim was to evaluate whether certain complications are missing or whether the one classification leads to inclined results compared with the other classification.

### Statistical Analysis

For classifying and describing different postoperative outcomes, both the GCCG and ECCG lists of complications were used separately. Both lists were presented and reported with outcomes 90 days after surgery. Both patient and tumor characteristics as well as complications grouped by the GCCG and ECCG classifications are presented as frequencies and percentages (Table 1). Comparison of differences between complications in the two classifications was performed using the McNemar test. For data management and analysis, SPSS version 27 (IBM, Armonk, NY, USA) was used.

**Table 1:** Characteristics of patients undergoing gastrectomy for gastric cancer in Finland during 2010–2016

	*n* = 1115 *n* (%)
Median year of surgery (IQR)	2013 (2011–2015)
Median age: years (IQR)	71 (63–79)
Sex	
Male	622 (55.8)
Female	493 (44.2)
CCI^a^	
0	512 (45.9)
1	361 (32.4)
2	144 (12.9)
≥ 3	98 (8.8)
Pathologic (yp/pTNM) stage	
0–I	282 (25.3)
II	303 (27.2)
III	388 (34.8)
IV	122 (10.9)
Missing	20 (1.8)
Surgical technique	
Open	1027 (92.1)
Laparoscopic	88 (7.9)
Type of gastrectomy	
Total	685 (61.4)
Distal	430 (38.6)
Resection	
Palliative intent	95 (8.5)
R2	82 (7.4)
R1	92 (8.3)
R0	801 (71.8)
Missing	45 (4.0)
Neoadjuvant treatment	
No	840 (75.3)
Yes	272 (24.4)
Missing	3 (0.3)

CCI, comorbidity classified with the Charlson Comorbidity Index^15^

## Results

From the registries, the study identified 1115 patients undergoing gastrectomy for gastric adenocarcinoma during 2010–2016 (Table 1). The median age at the time of operation was 71 years, and most of the patients underwent an open gastrectomy with curative intent.

### Complications

Complication developed for 257 (23.0%) of the patients (504 individual complications) according to the GCCG classification and for 483 (43.3%) of the patients (1084 individual complications) according to the ECCG classification, for an absolute difference of 226/1115 patients (20.3 percentage points). According to the GCCG classification, the most common complications were infections with both symptoms and germ isolation (8.4%), other major complications requiring re-intervention or other invasive procedures (5.1%), and anastomotic leak (3.9%). According to the ECCG classification, the most common complications were pneumonia (13.1%), intra-abdominal abscess (8.4%), and other infections requiring antibiotics (6.3%).

The incidences of complications with similar definitions are presented in Table 2. Complications with major differences between the GCCG and ECCG classifications are presented in Table 3. Notable differences in certain complication types between the classifications resulted in lower incidences of cardiac dysrhythmia (97% lower incidence; *p* < 0.001), infections (81% lower incidence; *p* < 0.001), and myocardial infarction (50% lower incidence; *p* = 0.008) using the GCCG classification.

**Table 2:** Occurrence of complications with similar definitions in the GCCG and ECCG classifications

Patients (*n* = 1115)	*n* (%)
Need for CPR	6 (0.5)
Pulmonary embolism with symptoms confirmed by urgent CT scan	19 (1.7)
Respiratory failure requiring reintubation	20 (1.8)
Pleural effusion requiring drainage	66 (5.9)
Pneumothorax requiring treatment	2 (0.2)
Acute liver dysfunction (the Child-Pugh score >8 for longer than 48 h)	11 (1.0)
Postoperative pancreatitis diagnosed both clinically and radiologically	4 (0.4)

CPR, cardiopulmonary resuscitation; CT, computed tomography

**Table 3:** Complications with major differences between the GCCG and ECCG classifications and the percentage of complications captured by the GCCG classification versus the ECCG classification

GCCG	*n* (%)	ECCG	*n* (%)	Difference (%)	*p* Value
Stroke causing patient’s permanent deficit	4 (0.4)	Stroke (CVA)	7 (0.6)	43	0.250
Myocardial infarction with patient’s transfer to CCU/ICU/other critical care facility	8 (0.7)	Myocardial infarction	16 (1.4)	50	0.008
Total dysrhythmias, including	1 (0.1)	Total dysrhythmias, including	31 (2.8)	97^a^	<0.001
Cardiac dysrhythmia requiring invasive treatment	1 (0.1)	Dysrhythmia atrial requiring treatment	29 (2.6)		
		Dysrhythmia ventricular requiring treatment	2 (0.2)		
Acute myocardial failure with acute pulmonary edema or drop in EF >50%	23 (2.1)	Congestive heart failure requiring treatment	40 (3.6)	43	<0.001
Total renal insufficiency, including	14 (1.3)	Total renal insufficiency, including	14 (1.3)	0^a^	1.000
Acute renal insufficiency (postoperative creatinine twice its preoperative value)/renal failure requiring CVVH or dialysis	14 (1.3)	Acute renal insufficiency (defined as doubling of baseline creatinine)	14 (1.3)		
		Acute renal failure requiring dialysis	3 (0.3)^b^		
Total infections (gastrointestinal, respiratory, urinary, or other) with both symptoms and germ isolation:	79 (7.1)	Total infections (any of the following):	422 (37.8)	81^a^	<0.001
Gastrointestinal	22 (2.0)	*Clostridium difficile* infection	10 (0.9)		
Respiratory	8 (0.7)	Intra-abdominal abscess	94 (8.4)		
Urinary	7 (0.6)	Wound infection requiring opening wound or antibiotics	37 (3.3)		
Other	10 (0.9)	Pneumonia	146 (13.1)		
Multiple	32 (2.9)	Intrathoracic abscess	5 (0.4)		
		Urinary tract infection	24 (2.2)		
		Central IV line infection requiring removal or antibiotics	6 (0.5)		
		Generalized sepsis	30 (2.7)		
		Other infections requiring antibiotics	70 (6.3)		
Postoperative bleeding requiring both urgent transfusions and invasive treatment	25 (2.2)	Gastrointestinal bleeding requiring intervention or transfusion	39 (3.5)	36	< 0.001
Total anastomotic or duodenal leaks, including	57 (5.1)	Total anastomotic or duodenal leaks, including	57 (5.1)	0^a^	1.000
Anastomotic leak (irrespective of presentation, method of identification, clinical consequences, or treatment)	44 (3.9)	(Esophagoentric) leak from anastomosis, staple line, or localized Conduit necrosis	57 (5.1)		
Duodenal leak (irrespective of presentation, method of identification, clinical consequences, or treatment)	13 (1.2)	Conduit necrosis/failure	0 (0.0)		
Other postoperative abnormal fluid from drainage and/or abdominal collections without gastrointestinal leak(s) preventing drainage removal or requiring treatment	56 (5.0)	Intra-abdominal abscess	56 (5.0)	0	1.000
Postoperative bowel obstruction (clinical/radiologic signs of obstruction, inability to enteral feed, longer need for NG suction)	61 (5.5)	Total ileus/bowel obstructions, including	61 (5.5)	0^a^	1.000
		Ileus defined as small bowel dysfunction preventing or delaying enteral feeding	49 (4.4)		
		Small bowel obstruction	12 (1.1)		
Delayed gastric emptying (by postoperative day10) requiring treatment or delaying discharge	17 (1.5)	Delayed conduit emptying requiring intervention or delaying discharge or requiring maintenance of NG drainage >7 days postoperatively	17 (1.5)	0	1.000
Total complications requiring re-interventions, including	62 (5.6)	Reoperation for reasons other than bleeding, anastomotic leak, or conduit necrosis	62 (5.6)	0^a^	1.000
Need for tracheostomy	1 (0.1)				
Postoperative bowel perforation or necrosis requiring surgical treatment (or cause of death)	4 (0.4)				
Other major complications requiring re-intervention or other invasive procedures	57 (5.1)				

GCCG, Gastrectomy Complications Consensus Group; ECCG, Esophagectomy Complications Consensus Group; CVA, cerebrovascular accident; EF, ejection fraction; CVVH, continuous veno-venous hemofiltration; IV, intravenous; NG, nasogastric

^a^Calculated based on total complications grouped due to similar or overlapping definitions

^b^All patients needing dialysis also had doubled creatinine.

The most common of the aforementioned complication types was infectious complications, with only 79 (8.4%) of the patients having infectious complications according to the GCCG classification, compared with 422 (37.8%) patients according to the ECCG classification (GCCG reported an 81% lower incidence; *p* < 0.001). The number of other reinterventions/reoperations was the same in the GCCG and ECCG classifications. Differences in definitions for reoperations led to inclusion of three endoscopic dilations for anastomotic stricture in the GCCG group, but not in the ECCG group, whereas three re-resections due to positive resection margins were included in the ECCG group, but not in the GCCG group.

Prolonged intubation and pancreatic fistula were reported only in the GCCG classification, recorded for 18 (1.6%) patients (Table 4). Finally, 20 separate types of complications were reported only in the ECCG classification, with 13 distinct types occurring in 131 instances (Table 5).

**Table 4:** Complications reported only according to GCCG classification

Complication	*n* (%)
Need for prolonged intubation (> 24 h after the surgical procedure)	10 (0.9)
Postoperative pancreatic fistula^14^	8 (0.7)

GCCG, Gastrectomy Complications Consensus Group

**Table 5:** Complications reported only according to ECCG classification

Complication	*n* (%)
Atelectasis mucous plugging requiring bronchoscopy	6 (0.5)
Acute aspiration	15 (1.4)
Acute respiratory distress syndrome	9 (0.8)
Chest tube maintenance for air leak for >10 days postoperatively	0 (0.0)
Pericarditis requiring treatment	0 (0.0)
Feeding J-tube complication	7 (0.6)
Pyloromyotomy/pyloroplasty complication	0 (0.0)
Urinary retention requiring reinsertion of urinary catheter, delaying discharge, or discharge with a urinary catheter	15 (1.4)
Deep venous thrombosis	2 (0.2)
Peripheral thrombophlebitis	0 (0.0)
Recurrent nerve injury	1 (0.1)
Other neurologic injury	7 (0.6)
Acute delirium	20 (1.8)
Delirium tremens	0 (0.0)
Thoracic wound dehiscence	0 (0.0)
Acute abdominal wall dehiscence/hernia	29 (2.6)
Acute diaphragmatic hernia	1 (0.1)
Chyle leak	11 (1.0)
Multiple organ dysfunction syndrome	8 (0.7)

## Discussion

In this population-based nationwide study, the occurrence of postoperative complications 90 days after surgery according to the GCCG classification definitions was 23%, compared with 43% according to the ECCG definitions, suggesting that half of complications are missed by the GCCG classification. Furthermore, 2% of the patients had complications recorded only in the GCCG classification, whereas 16% had complications recorded only in the ECCG classification.

The main strengths of this study were its unselected population-based nationwide design and the large size of the cohort. The study design was retrospective, which could be considered a weakness, especially considering the collection of complication data. However, the published estimates of complication incidence from FINEGO suggest complication estimates comparable with those of the prospective Dutch national cohort based on the ECCG definitions.^12,13^ For the current comparison, the patient records were re-reviewed to evaluate the complications according to the definitions of the GCCG. However, some complication categories still may have misclassified data compared with a prospective database.

The occurrence of complications in the current study according to the GCCG classification was relatively similar to that of the Italian GASTRODATA study, which estimated the incidence of postoperative complications at 30%.^10^ Furthermore, no difference in the occurrence of total complications was observed between this current study and the DUCA study using the ECCG classification.^5^ As evident from the results, GCCG classification is focused, although inconsistently, on major complications, resulting in an underestimation of the minor complications. Furthermore, the GCCG classification in many categories requires certain treatments or sequelae for complications (e.g. with strokes), resulting in underestimates of incidences. For example, a major stroke successfully treated with thrombectomy and without sequelae would not qualify for stroke in the GCCG classification, although many would consider this a major complication. Even if a patient does not get a permanent deficit, a stroke is a clear setback in recovery from surgery requiring additional effort from caregivers, and the hospital stay may be lengthened. However, in the Clavien-Dindo classification a postoperative stroke is classified as a grade IV complication and therefore considered a major complication, even if the patient experiences no neurologic deficit.

Cardiac dysrhythmias are recorded in the GCCG only if invasive treatment is required. Although atrial fibrillation may have little direct effect on the patient, many are put on an anticoagulant medication, which has its own risks and economic impacts. Similarly, the requirement of an objectively decreased ejection fraction (i.e., echocardiography for recording acute myocardial failure or germ isolation for recording pneumonia) in the GCCG is a likely reason for lower incidences of complications compared with the ECCG. In clinical practice, pneumonia is often treated based on clinical symptoms and radiologic findings without obtaining bronchoalveolar lavation samples. The GCCG classification also overlooks minor bleeding requiring transfusions or minor infections requiring antibiotics. However, even these more minor events might lengthen the hospital stay and have an economic impact on society. Taken together, using commonly accepted definitions for certain events, such as the American Thoracic Society criteria for pneumonia or WHO criteria for myocardial infarction in the GCCG, would further improve the comparability of complication data collected using the GCCG framework.

The ECCG classification identified four sentinel complications that afterward can be further classified according to their resource utilization. This has not been done in the GCCG, although the more specific evaluation of at least anastomotic and duodenal stump leak would be beneficial in research and the clinical context of gastric cancer surgery. Furthermore, as shown in Table 5, several types of complications with occurrence of at least 1% are not recorded using the GCCG classification but are included in the ECCG classification. The GCCG classification would benefit from a revision including some of these relevant complications, such as aspiration and abdominal wall dehiscence.

This study has several future implications. First, using the ECCG compared with the GCCG complication classification seems preferable due to a more comprehensive list of relevant complications and a higher sensitivity of complications. However, in the gastrectomy context, adding duodenal leaks to the ECCG complications to differentiate between the two distinct types of leaks (anastomotic and duodenal stump) is reasonable. The GCCG classification could be further criticized for its lack of clear definitions for some key outcomes, such as escalation of care, blood product utilization, and quality of life data. Adding more specific definitions to the outcome measures would benefit the GCCG classification.

Finally, because some cancers in the esophagogastric junction can be treated with either esophagectomy or gastrectomy, a uniform classification for both types of resections would be preferable. Even if the differences between the two classifications might not have a direct impact on perioperative management or interventions, having a standardized and established consensus for reporting postoperative complications could enable comparison of the complication profiles between other upper GI-tract surgeries.

In conclusion, the current population-based study suggests that the ECCG classification is more comprehensive and sensitive in evaluating complications of gastrectomy than the GCCG classification. Using the ECCG classification may be preferable in the context of gastrectomy.

## Data Availability

The data can be shared for research purposes upon request by contacting the Chief Investigator, Professor Joonas Kauppila, but may be restricted by and require complimentary permissions from the ethical committee and relevant original data holders.
